# Empirical Power and Sample Size Calculations for Cluster-Randomized and Cluster-Randomized Crossover Studies

**DOI:** 10.1371/journal.pone.0035564

**Published:** 2012-04-27

**Authors:** Nicholas G. Reich, Jessica A. Myers, Daniel Obeng, Aaron M. Milstone, Trish M. Perl

**Affiliations:** 1 Division of Biostatistics and Epidemiology, University of Massachusetts, Amherst, Massachusetts, United States of America; 2 Division of Pharmacoepidemiology and Pharmacoeconomics, Brigham and Women’s Hospital, Boston, Massachusetts, United States of America; 3 Department of Biostatistics, Johns Hopkins Bloomberg School of Public Health, Baltimore, Maryland, United States of America; 4 Department of Pediatrics, Division of Pediatric Infectious Diseases, Johns Hopkins University School of Medicine, Baltimore, Maryland, United States of America; 5 Department of Medicine, Division of Infectious Diseases, Johns Hopkins University School of Medicine, Baltimore, Maryland, United States of America; Vanderbilt University, United States of America

## Abstract

In recent years, the number of studies using a cluster-randomized design has grown dramatically. In addition, the cluster-randomized crossover design has been touted as a methodological advance that can increase efficiency of cluster-randomized studies in certain situations. While the cluster-randomized crossover trial has become a popular tool, standards of design, analysis, reporting and implementation have not been established for this emergent design. We address one particular aspect of cluster-randomized and cluster-randomized crossover trial design: estimating statistical power. We present a general framework for estimating power via simulation in cluster-randomized studies with or without one or more crossover periods. We have implemented this framework in the clusterPower software package for R, freely available online from the Comprehensive R Archive Network. Our simulation framework is easy to implement and users may customize the methods used for data analysis. We give four examples of using the software in practice. The clusterPower package could play an important role in the design of future cluster-randomized and cluster-randomized crossover studies. This work is the first to establish a universal method for calculating power for both cluster-randomized and cluster-randomized clinical trials. More research is needed to develop standardized and recommended methodology for cluster-randomized crossover studies.

## Introduction

Clinical trials are often designed to assess the effectiveness of a particular intervention. While evidence from individually-randomized, masked clinical trials is considered the gold standard of scientific evidence, in many settings, such a design is not feasible and, sometimes, is unethical. Cluster-randomized trials randomize groups of people instead of individuals. These studies can be valuable tools for evaluating interventions that are best implemented at the group level. Some have argued that a cluster-randomized design yields more accurate estimates of the treatment effect of interest because the treatment effect is estimated on the level at which the intervention is applied [1]. Looking forward, cluster-randomized designs will continue to play an important role in clinical effectiveness research, filling in when individually randomized studies are not possible.

Many questions remain about best practices for cluster-randomized studies. A variant to the cluster-randomized study design, the cluster-randomized crossover design, has been touted as a methodological advance that can increase efficiency of cluster-randomized studies in certain situations. While the cluster-randomized crossover trial has become a popular tool, standards of design, analysis, reporting and implementation have not been established for this emergent design. This is largely due to the fact that the principles from cluster-randomized trials with no crossover are not easily applied to a crossover setting. The crossover introduces a significant paradigm change in analyzing cluster-randomized data. In a cluster-randomized crossover trial, statistical inference is based on evidence drawn from within-cluster comparisons. In standard cluster-randomized trials, between-cluster comparisons provide the evidence. Therefore, techniques for analyzing data from cluster-randomized crossover trials are very different from those used to analyze data from cluster-randomized trials with no crossover.

In this paper, we discuss a single aspect of designing cluster-randomized and cluster-randomized crossover trials: estimating statistical power. Many scientific studies set out to gather evidence that can be used to evaluate a specific hypothesis. An investigator designing a study must examine carefully the likelihood that the study will produce definitive evidence for or against a hypothesis. Statistical power is defined as the probability of correctly rejecting the null hypothesis, or phrased another way, the probability of having conclusive evidence for one hypothesis over another given the existing study design. Accurate power calculations provide guidance for the appropriate sample-size requirement for a given study. Obtaining an accurate estimate of the necessary sample size to answer a given scientific question in a particular setting ensures that researchers do not enroll too few or too many participants in a study. These calculations, a vital part of any thorough study proposal, can be complicated in cluster-randomized settings because of the correlation structures that are often present within clustered data.

Methods for calculating power for cluster-randomized trials exist in patchwork, with investigators having to hunt for the method or equation that fits their specific needs. Formulas do exist to calculate power for cluster-randomized trials with continuous or dichotomous outcomes [2] or for cluster-randomized trials with continuous, dichotomous or categorial outcomes [3], however they cannot easily be generalized to incorporate a crossover period. Methods to analyze cluster-randomized crossover trials with continuous or dichotomous outcomes have been proposed [4]–[6], as have methods for calculating power in cluster-randomized crossover settings with continuous outcome data [7]. Although these methods have been developed for specific cluster-randomized designs, no unified framework has enabled power calculations in both cluster-randomized and cluster-randomized crossover settings. We present such a framework which enables apples-to-apples comparisons between the two study designs and can be important for an investigator wishing to compare the efficiency of the two designs.

For complex study designs, such as the cluster-randomized crossover, simple formulas to calculate power may not adequately capture the expected variability from observed data. In these cases, estimation of power via simulation methods may be needed [8]. Simulation methods have the added advantage of being able to work in a wide range of settings, both simple and complex. This can facilitate comparisons between study designs, such as those presented in Example D, below. We present a unifying data generating model for continuous, dichotomous or count outcome data from a cluster-randomized study – with or without one or more crossover periods. This framework is implemented in the clusterPower software package for R, which has been designed to run simulated power calculations. We simulate an empirical estimate of the power that can be used by researchers in the study design phase. This tool is, to our knowledge, the only freely available tool that can calculate power for a wide range of standard cluster-randomized and cluster-randomized crossover study designs.

In the following Methods section we present our software tool and discuss the underlying data generating model. In the Results section we give three examples of the tool in practice. In the Discussion section we address possible limitations and extensions of this work.

## Methods

### Overview

Simulation is a powerful tool for estimating the power of a complex study when the data analysis procedure is not straight-forward (see, for example, [9], p. 176 or [10]). Indeed, one paper has discussed the idea of simulation of power for cluster-randomized trials, although only in the context of continuous data [11]. The idea is to randomly generate numerous datasets, each of which represents a hypothetical version of the study to be conducted. The datasets are generated assuming that a specific alternative hypothesis is true. The null hypothesis is that a treatment has no effect 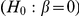 and the alternative is that the treatment has an effect 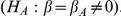 As with other types of power calculations, a specific alternative treatment effect is specified – for example, 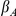 could equal 2 – and datasets are generated from the resulting model. For each of these datasets, the data analysis is carried out and the evidence for or against the null hypothesis is recorded. A Type-I error rate, commonly referred to as 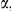 is the probability of rejecting a null hypothesis when the null hypothesis is actually true. Typically, biomedical researchers set an acceptable 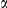-level at 1 in 20, or 0.05. In our example, if 1000 datasets are generated and in 800 of them the null hypothesis is correctly rejected (i.e. the p-value is less than 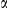), then we would estimate an empirical power of 800/1000 or 80%.

We have developed free software, the clusterPower package for R, which generates simulated datasets as described above and analyzes each dataset using a particular statistical method which can be customized for the situation [12], [13]. The clusterPower package can be downloaded for free from the Central R Archive Network (CRAN) or from github, a popular code collaboration site. In addition, making the source code available on github allows for anyone with necessary skills and interest to offer additions and/or improvements to the existing package. The functions power.sim.normal(), power.sim.binomial() and power.sim.poisson() return the estimates of treatment effect as well as the empirical power estimate for the particular parameter combination used to simulate the data. To our knowledge, no other free software is available to calculate power for cluster-randomized crossover trials with all of these three types of outcome data.

### Data Generating Model

We have a study with 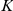 clusters, 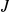 study periods and 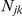 participants in the 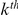 cluster and the 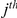 period. We define 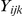 as the random variable representing the outcome of interest for the 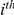 individual in the 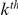 cluster during the 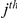 period of the study. The treatment assignments will vary by cluster and period. Therefore, we will use a separate indicator variable, 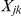 to indicate whether cluster 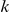 during period 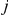 is assigned to the treatment arm 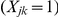 or the control arm 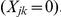 Cluster-randomized trials with no crossover will be treated as the subset of cluster-randomized crossover trials that have a single period of study. We assume a generalized linear mixed model (GLMM) framework of
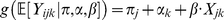
(1)where 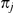 are fixed period effects, 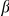 is the fixed treatment effect and the 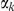 are random cluster effects following a normal distribution 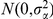.

This is a cluster-level model, as no individual-level characteristics appear as predictors or covariates in equation 1. In the design phase of a cluster-randomized crossover trial, it is common practice to power a study based on adjustment for only cluster-level variables, ignoring individual-level variability in covariates. Equation 1 is an example of such a model. It accounts for cluster-level correlation but does not introduce individual-level covariates – keeping the model at a manageable level of complexity for a simulation-based power calculation. We will consider parametric GLMMs for continuous, binary and count outcome data.

The overall goal is to simulate data from the general model so that an empirical power calculation can be run. Some of the data-generating parameters are the same for all types of data and others are not. The following subsections outline the other specific information needed to generate individual-level data from these models for analysis. Table 1 summarizes the parameters used in the data generating model, translating between the the notation used in this manuscript and the R code needed for implementation.

**Table 1:** Parameters from data generating models needed to simulate power.

notation	text	outcome data type		
		continuous	binary	count
*K*	n.clusters	✓	✓	✓
*J*	n.periods	✓	✓	✓
*N_jk_*	clust.size	✓	✓	✓
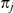 , 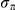	period.effecta, period.var b	✓	✓	✓
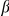	effect.size	✓	✓	✓
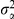	btw.clust.var	✓	✓	✓
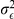	indiv.varc	✓		
ICC	ICCc	✓		
*T_ijk_*	at.risk.params			✓

a The period effects are drawn from a normal distribution centered at period.effect with variance period.var.

b If period.var = 0, then period.effect is assumed to be the same for all periods.

c Only one of the ICC and needs to be specified in continuous data generating models.

#### Continuous outcome data

Continuous outcome data will be generated using a Gaussian or normal GLMM, also known as a linear mixed model. In this setting, 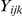 is assumed to be a continuously measured outcome variable. This model will be of the form
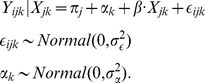
(2)


Data can be simulated from this model by specifying the fixed effects (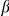 and 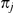) and distributions for the random effects. The outcome is modeled on the same scale as the predictor function, so the fixed effects are specified on the same scale as the outcome measurements. If we define the intraclass correlation coefficient ICC = 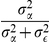, then specifying two of the three parameters (ICC, 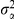, ) is sufficient to simulate observations from the model.

#### Binary outcome data

Binary data will be generated using a logistic GLMM. Here, 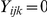 or 1, depending on how the binary outcome is defined. We model the probability of the outcome as
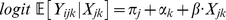
(3)

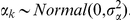



By assuming values for the fixed effects and specifying the variance of the random cluster effects, 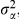 we can simulate data from this model. Care must be taken in specifying the variance, and input from experts and/or past studies is vital.

#### Poisson outcome data

Count data will be generated using a log-linear Poisson GLMM. In this setting, 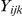 is assumed to be a non-negative integer and 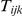 is the at risk time for person 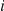 in group 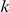 during period 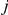. This model will be of the form
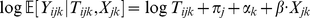
(4)

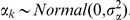



By assuming values for the fixed effects and specifying the variance of the random cluster effects, 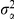, we can simulate data from this model. Care must be taken in specifying an appropriate variance, and input from experts and/or past studies is vital. For simple power calculations, one may assume 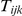 to be the same for all individuals. However, we can simulate different at-risk times for participants by specifying parameters for a negative binomial distribution. For example, we can assume that 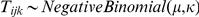 where the mean of this distribution is 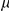 and the variance is 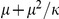. In this parameterization the parameter 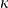 is referred to as the size or dispersion of the distribution. Using this formulation for exposure times allows the researcher to adjust the variability in the exposure times to fit the application of interest.

## Results

This section presents three examples of the clusterPower package. The R code for all examples is available as Code S1.

### Example A: A Cluster-randomized Crossover Trial with No Period Effect

We developed this hypothetical example of a power calculation based on our experience with the Pediatric SCRUB clinical trial, a multi-center, cluster-randomized crossover trial. A nested study from the SCRUB trial has been described elsewhere [14]. This trial aims to evaluate the effectiveness of daily clorhexidine gluconate (CHG) bathing in pediatric intensive care units (PICUs) to reduce blood-stream infections. The actual study design and analysis plan differed slightly from what we present here, which we have simplified for the purposes of illustration. Ten PICUs participated in this study. Subsequently, each PICU participated for two six-month study periods separated by a two-week washout period. Five PICUs were randomly chosen to receive CHG treatment in the first time period and standard of care in the second. The other five were assigned the reverse treatment ordering: first standard of care, then CHG. For the duration of the entire study, active surveillance registered blood-stream infection events and each enrolled patient’s time at risk was recorded.

We used equation 4 to simulate data from hypothetical realizations of this trial. Each cluster-period had 210 participants and each participant was assumed to have 10 at-risk days. In reality the at-risk days vary widely by individual. However our calculations were aggregated at the cluster level since we assume that there are no individual-level risk factors. Therefore, we assumed that each cluster accumulates 2100 at-risk days per period, or over 300 at-risk days per month. This was roughly in line with observed data from the pilot and study period.

Further, we assumed that the baseline risk of blood-stream infection was 4 infections per 1000 person at-risk days and did not change with study period, i.e. 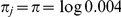 for all 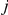. The treatment was assumed to reduce the risk of blood-stream infection by 25%, corresponding to a relative risk estimate of 0.75. Therefore, we set 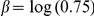. For each dataset, 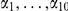 were drawn from a normal distribution centered at zero with variance 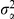. The baseline incidence rate and variance were specified with input from clinical experts and by consulting past published data [15]. We chose the variance (with precision to the nearest tenth of a decimal place) so that 9 out of every 10 clusters would have infection rates below 10 infections per 1000 at-risk days. For our primary power calculation, 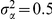 met this criteria.

We used a fixed effects Poisson regression model to draw inference about the treatment effect. The model was fit to the 20 datapoints (two observations for each of ten units). In this approach, inference was drawn about the parameter 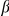 based on whether the 95% confidence interval covered zero; if the confidence interval covered zero then we failed to reject the null hypothesis.

For the fixed effects Poisson regression model, we assumed that
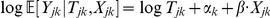
where 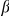 is an estimate of the treatment effect and the 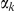 are cluster-specific parameters for 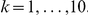 In this model, we have 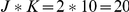 observations, and we fit 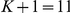 parameters. The 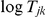 are used as an offset.

For this example, power may be calculated using the power.sim.poisson() function in the clusterPower package for the statistical software R using a single command. The lines of code below demonstrate how the package may be freely downloaded and installed from the internet, loaded into the a working R environment, and run. The set.seed(17) command sets the random seed to a fixed number, ensuring that the results shown here are reproducible.

>install.packages(“clusterPower”)

>library(clusterPower)

>set.seed(17)

>p<-power.sim.poisson(n.sim = 1000, effect.size = log(.75), alpha = .05,

n.clusters = 10, n.periods = 2, cluster.size = 210,

btw.clust.var = .5, at.risk.params = 10,

period.effect = log(.004), period.var = 0,

estimation.function = fixed.effect.cluster.level)

>p$power

[1] 0.508

Full documentation of the power.sim.poisson() command is available online and in the help files within R.

Table 2 shows one of the simulated datasets, including the crude estimates of the incidence rate ratio within each cluster. The results of our power simulation example, which simulated 1000 such datasets, show that in this setting there is just over 50% power to detect a 25% reduction in the relative risk of infection due to the CHG intervention.

**Table 2:** One of the 1000 Simulated data sets.

	number of events		
unit	control	treatment	IRRa
1	14	10	0.71
2	17	7	0.41
3	8	3	0.38
4	6	4	0.67
5	11	5	0.45
6	20	7	0.35
7	12	15	1.25
8	5	5	1.00
9	4	4	1.00
10	9	8	0.89

a The incidence rate ratio (IRR) is the number of treatment events divided by the number of control events.

**Figure 1:**
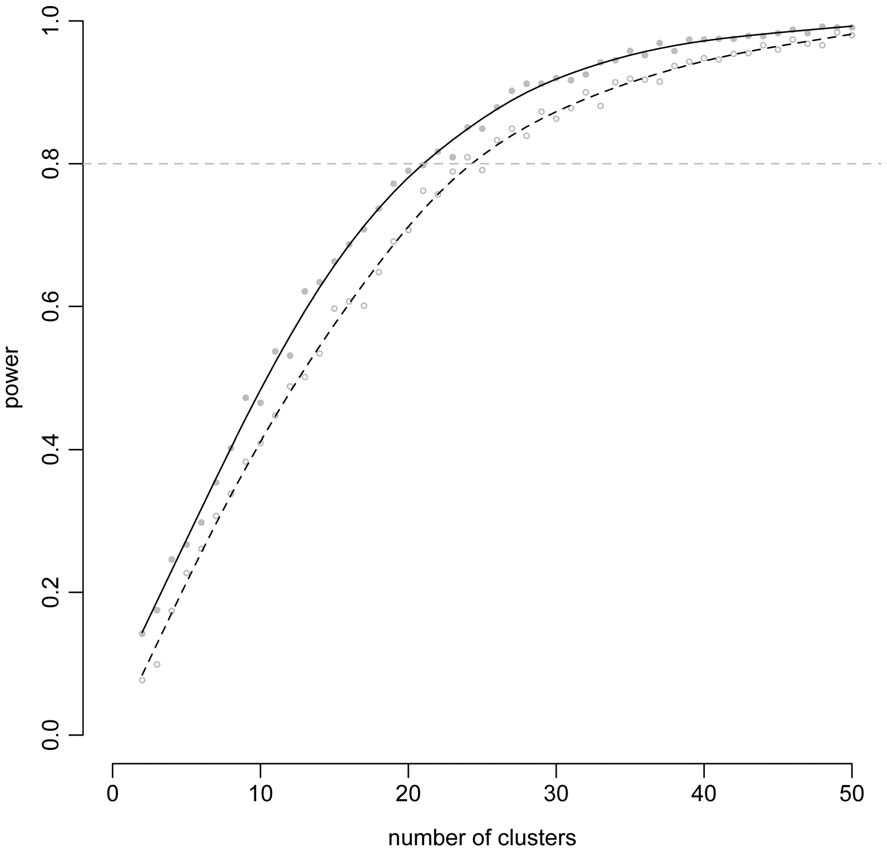
Power curves from Examples A and B. These curves show the relationship of power with the number of clusters. The points show simulated power for 1000 datasets with a smoothed line drawn through the data to highlight the overall pattern. The solid line and gray points represent the simulations with constant baseline rates (Example A) and the open circles and dashed line represent the simulations with time-varying baseline rates (Example B).

Additionally, this one-off power calculation could be supplemented with an exploration of power as different parameters – for example, the number of clusters, overall sample size or between-cluster variability – change. To illustrate such a use, we simulated power for our study across a range of cluster-sizes while keeping our earlier assumptions. Figure 1 shows that to achieve 80% power in a study with 210 participants per cluster-period, 22 clusters would be needed. These types of additional simulations can give more insight into the appropriate study design and can inform a final authoritative power calculation.

### Example B: A Cluster-randomized Crossover Clinical Trial with Time-varying Prevalence

We extend the previous example to incorporate time-varying incidence rates of blood-stream infections. Here, we assume that the background incidence rate of blood-stream infections is decreasing over time. In the first period of the study we assume that the background rate is 4 infections per 1000 person at-risk days and in the second period we assume that it is 3 infections per 1000 person at-risk days. We fit a model that includes a fixed period effect, so our estimate of the treatment effect is adjusted for this secular trend.

In this scenario, 24 clusters are needed to obtain 80% power. This is two more than the 22 needed in the scenario with a fixed background rate. Figure 1 compares the simulated power of the two scenarios, and we observe a clear and quantifiable drop in power when the time-varying period effect is included. Code to reproduce example B is available in the Code S1.

### Example C: A Cluster-randomized Incentive Trial

We now present a power calculation for a proposed trial of incentives to improve medication adherence. In this trial, investigators are collaborating with a large pharmacy benefits provider to randomize patients to either a new benefit design (including reduced co-pays) or control. Due to ethical and trial considerations, randomization must take place at the employer health plan level. The number of employer health plans available to be randomized is fixed at 8, but we may control the size of the sample within each employer. The outcome, adherence to a prescribed preventive cardiovascular medication, will be measured as a binary variable for each patient. We expect the adherence rate in the control group to be approximately 50% and the variation in adherence rates across clusters to be very small 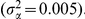


**Figure 2:**
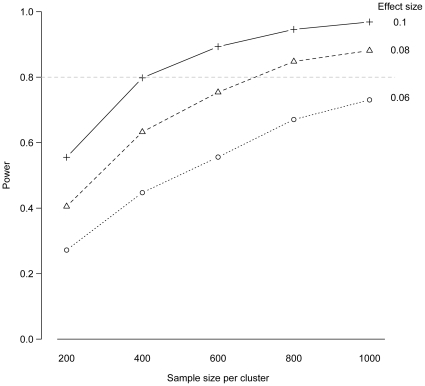
Power curves from Example C. These curves depict the relationship between power and sample size per cluster across different effect sizes. The points show simulated power for 500 datasets.

We considered power under varying effect sizes (differences in adherence between intervention and control arms of 0.06, 0.08, and 0.1). We also varied the number of patients sampled per cluster. We used the power.sim.binom() function to simulate 500 datasets for each set of parameter values. Because the number of clusters is low, power was calculated assuming that a simple fixed effects logistic regression model will be applied for data analysis. Figure 2 shows the power calculated under all parameter values with sample size on the x-axis and lines connecting data points calculated under the same effect size. Under a true difference in adherence of only 6%, a high power cannot be achieved with even the highest sample size considered. If the effect size is 8% or larger, then 1000 patients per cluster will be sufficient to achieve 80% power. Code to reproduce example C is available in the Code S1.

### Example D: A Clinical-effectivenes Cluster-randomized Crossover Trial

Hejblum et al. (2009) conducted a cluster-randomized crossover trial in 21 intensive care units in France to examine the change in the number of chest radiographs taken when comparing the status quo to a new on-demand strategy for ordering the radiographs. The results in the paper suggest that the on-demand strategy for ordering chest radiographs could significantly reduce the number of chest radiographs taken without impacting quality of care. To show the value the crossover design provides to a study such as this one, we calculated power for two hypothetical cluster-randomized studies – one with a crossover and one without – that attempt to replicate the findings of Hejblum et al.

We assumed that the average amount of time a patient spent on mechanical ventilation was 5 days. Also based on the published data, we assumed that an average of 1 chest radiographs per day were taken while on mechanical ventilation in the status quo group. We ran these calculations, assuming a type-I error rate of 5% and assuming a between-cluster variance of 0.01 (chest-radiographs per day)^2^. The code to run this analysis is given below:

>n.clusters <-20

>size <-20

>nsim <-1000

>bcv <-.01

>at.risk <-5

>baseline <-1

>exD.crxo <-power.sim.poisson(n.sim=nsim, effect.size=log(.9), alpha=.05,

n.clusters=n.clusters, n.periods=2,

cluster.size=size, btw.clust.var=bcv,

at.risk.params=at.risk, verbose=FALSE,

period.effect=log(baseline), period.var=0,

estimation.function=fixed.effect.cluster.level)

>exD.crxo$power

[1] 0.912

>exD.cr <—power.sim.poisson(n.sim=nsim, effect.size=log(.9), alpha=.05,

n.clusters=n.clusters, n.periods=1, cluster.size=size*2,

btw.clust.var=bcv, at.risk.params=at.risk, verbose=FALSE,

period.effect=log(baseline), period.var=0,

estimation.function=fixed.effect.cluster.level)

>exD.cr$power

[1] 0.336

For the hypothetical cluster-randomized crossover study with 20 clusters of 20 participants in each period (800 participants total), the study would have 91.2% power to detect a 10% reduction in the number of chest radiographs ordered. For the hypothetical cluster-randomized study with 20 clusters and 40 participants in each cluster (and no crossover period) we would have only 33.6% power to detect the same 10% reduction. We see that in a study such as this, the crossover design plays a crucial role in making a study viable.

## Discussion

This manuscript introduces a practical and freely-available tool for researchers to run power calculations for cluster-randomized and cluster-randomized crossover studies. By providing a standardized platform for calculating power for these types of studies, this tool – the clusterPower package for R – enables comparisons between the two types of designs and may aid researchers in developing efficient study protocols. Cluster-randomized studies have become increasingly popular in recent years. In fields such as hospital epidemiology and educational research, they are commonplace. The benefits and drawbacks to a cluster-randomized design are increasingly being discussed [1], [16], [17]. However, as this design and its derivatives (such as the cluster-randomized crossover design) become more widely used, a more thorough understanding of optimal settings for these designs is crucial. In particular, cluster-randomized crossover studies provide a unique design for evaluating the clinical effectiveness of a particular intervention.

While our software makes a complicated calculation accessible and easy to implement, careful attention is required to ensure that the inputs for the simulator are accurate. Determining the appropriate between-cluster variance may not be a trivial task, especially if background data is limited or does not exist. Furthermore, our software is designed for a general and relatively simple scenario. Although we expect this design to cover many common designs, some studies may not fit these pre-packaged formulas. For studies with design complexities not covered by these settings, additional programming may be necessary to use this framework. Using an individual-level framework for simulation would create a more flexible data generation architecture, allowing for individual-level variability to be simulated. Currently, this has not been implemented in our framework.

The framework that we introduce in this manuscript creates opportunities to expand our knowledge about the dynamics of cluster-randomized studies. For example, increased between-cluster variability may lead to better or worse power, holding all other things equal. Similarly, the impact of increased variability of cluster sizes on power could be explored. Often, statisticians report a single number as a target enrollment size for all clusters. Power may change if all clusters had exactly the same size or if clusters had vastly different sizes. Additionally, the benefit achieved from adding a crossover to a cluster-randomized design is unknown. The software described in this manuscript provides a convenient platform to run tailored simulations to explore all of these scenarios. As future work, we would like to implement versions of these functions in other statistical software, like STATA or SAS.

There are some distinct benefits of the simulation framework presented here in comparison with other methods currently in use. First, this framework provides a consistent method that can be applied across a very wide range of cluster-randomized studies. Common types of outcome data are supported and crossover designs are incorporated with ease. Second, the analysis methods used here – GLMMs – are the methods that are often used in published analyses. And, if they do not match the statistical technique that will be used for a given study, our software can be extended by the end-user with straight-forward programming that provides a data analysis function with standardized inputs and outputs.

Some studies may wish for their final data analysis to include individual-level predictors, especially if some such predictors may account for a large portion of the variability in the outcome. However, in crossover studies, the design is explicitly meant to create balance on these potential confounders, and studies that have compared cluster- and individual-level analyses of cluster-randomized crossover studies have shown little difference [6].

While research has been done to determine optimal methods for analyzing continuous outcome data in cluster-randomized crossover trials [5], additional work is needed to establish the best methods for binary and count data.

As the use of cluster-randomized studies becomes more common, tools such as the one we introduce and demonstrate in this manuscript provide practical solutions to often intractable sample size calculations while furnishing a platform for gaining a deeper understanding of the dynamics of cluster-randomized and cluster-randomized crossover trials.

## Supporting Information

Code S1 **The R code for all examples.**
(R) Click here for additional data file.
